# Laparoscopic Low Anterior Resection with Ureterectomy and Ileal Ureter Interposition for Ureter-Invading Locally Recurrent Rectal Cancer: A Case Report

**DOI:** 10.70352/scrj.cr.26-0394

**Published:** 2026-09-01

**Authors:** Takumi Yamamoto, Yusuke Kitagawa, Hisanori Miki, Jun Watanabe, Yosuke Fukunaga

**Affiliations:** 1 Colorectal Surgery, Kansai Medical University Medical Center, Moriguchi, Osaka, Japan; 2 Colorectal Surgery, Kansai Medical University, Moriguchi, Osaka, Japan

**Keywords:** locally recurrent rectal cancer, pelvic sidewall recurrence, ureteral invasion, ileal ureter interposition, laparoscopic surgery, renal preservation, nephrostomy

## Abstract

**INTRODUCTION:**

Locally recurrent rectal cancer (LRRC) with ureteral invasion is surgically demanding because curative-intent resection often requires en bloc ureterectomy, and urinary diversion may be considered. Although ileal interposition for ureteral reconstruction can preserve renal function when direct ureterocystostomy or uretero-ureterostomy is not possible, reports of this procedure for LRRC remain scarce.

**CASE PRESENTATION:**

An 81-year-old man underwent laparoscopic high anterior resection for rectosigmoid adenocarcinoma (pT3N1bM0, pStage IIIB) in March 2020, and adjuvant chemotherapy was not administered because of the patient’s preference. Twenty months later, a metachronous liver metastasis was detected and resected by laparoscopic partial hepatectomy. Surveillance imaging in 2023 revealed an approximately 20-mm tumor consistent with recurrence located anterior to the left iliac bifurcation, involving the left ureter at the common iliac crossing and causing hydronephrosis, for which percutaneous nephrostomy was performed for decompression. The tumor was RAS-mutant, BRAF wild-type, and microsatellite stable. FOLFOX plus bevacizumab was selected as a standard systemic treatment option for recurrent colorectal cancer with RAS mutation, but the treatment was discontinued due to toxicity, mainly fatigue. Because the disease remained confined to the pelvis without distant metastasis on PET/CT and first-line chemotherapy became difficult to continue, curative-intent resection was planned. Curative-intent laparoscopic low anterior resection with en bloc ureterectomy was performed. Because tension-free ureterocystostomy or uretero-ureterostomy was considered difficult due to limited tissue mobility, a short-segment isoperistaltic ileal ureter interposition was constructed. Postoperative paralytic ileus resolved conservatively (Clavien–Dindo grade II). The urinary catheter, ureteral stent, and nephrostomy tube were removed on PODs 8, 32, and 49, respectively, without deterioration of renal function. No adjuvant therapy was administered. The patient remained free of clinical and radiological recurrence 12 months after surgery.

**CONCLUSIONS:**

Laparoscopic salvage resection with en bloc ureterectomy and ileal ureter interposition may be a feasible renal-preserving option for selected patients with ureter-invading LRRC.

## Abbreviations

CA19-9
: carbohydrate antigen 19-9
CEA
: carcinoembryonic antigen
FEEA
: functional end-to-end anastomosis
FOLFOX
: oxaliplatin, leucovorin, and 5-fluorouracil
LRRC
: locally recurrent rectal cancer

## INTRODUCTION

LRRC remains one of the most technically demanding conditions in colorectal surgery, because durable oncologic control largely depends on achieving an R0 resection in a pelvis distorted by prior operations.^1)^ When recurrence involves the ureter, curative-intent surgery often requires en bloc ureterectomy. In such settings, urinary diversion may be considered; however, it is associated with substantial perioperative morbidity and may impose long-term QOL impairment.^2)^

Ileal ureter interposition is an established reconstructive option for long ureteral defects and can facilitate renal preservation when tension-free ureterocystostomy is not feasible. Nevertheless, detailed descriptions of ileal ureter interposition in a colorectal surgery–focused context are uncommon, particularly for LRRC, where severe adhesions and distorted pelvic anatomy after previous surgery increase technical complexity.

Moreover, although minimally invasive approaches have been selectively adopted for salvage pelvic surgery, reports of laparoscopic salvage rectal resection combined with concomitant ureterectomy and ileal ureter interposition remain scarce in the colorectal literature.

Here, we report a case of ureter-invading LRRC treated with laparoscopic salvage low anterior resection with a diverting stoma, en bloc ureterectomy, and short-segment isoperistaltic ileal ureter interposition. This report highlights key technical considerations for renal-preserving urinary reconstruction in the salvage setting and perioperative management strategies relevant to urologic anastomoses.

## CASE PRESENTATION

An 81-year-old man underwent laparoscopic high anterior resection for rectosigmoid adenocarcinoma 3 years earlier. Pathological staging was pT3N1bM0, pStage IIIB. He declined adjuvant chemotherapy. One year later, metachronous liver metastasis was detected, and laparoscopic partial hepatectomy of segment 8 was performed.

**Fig. 1:**
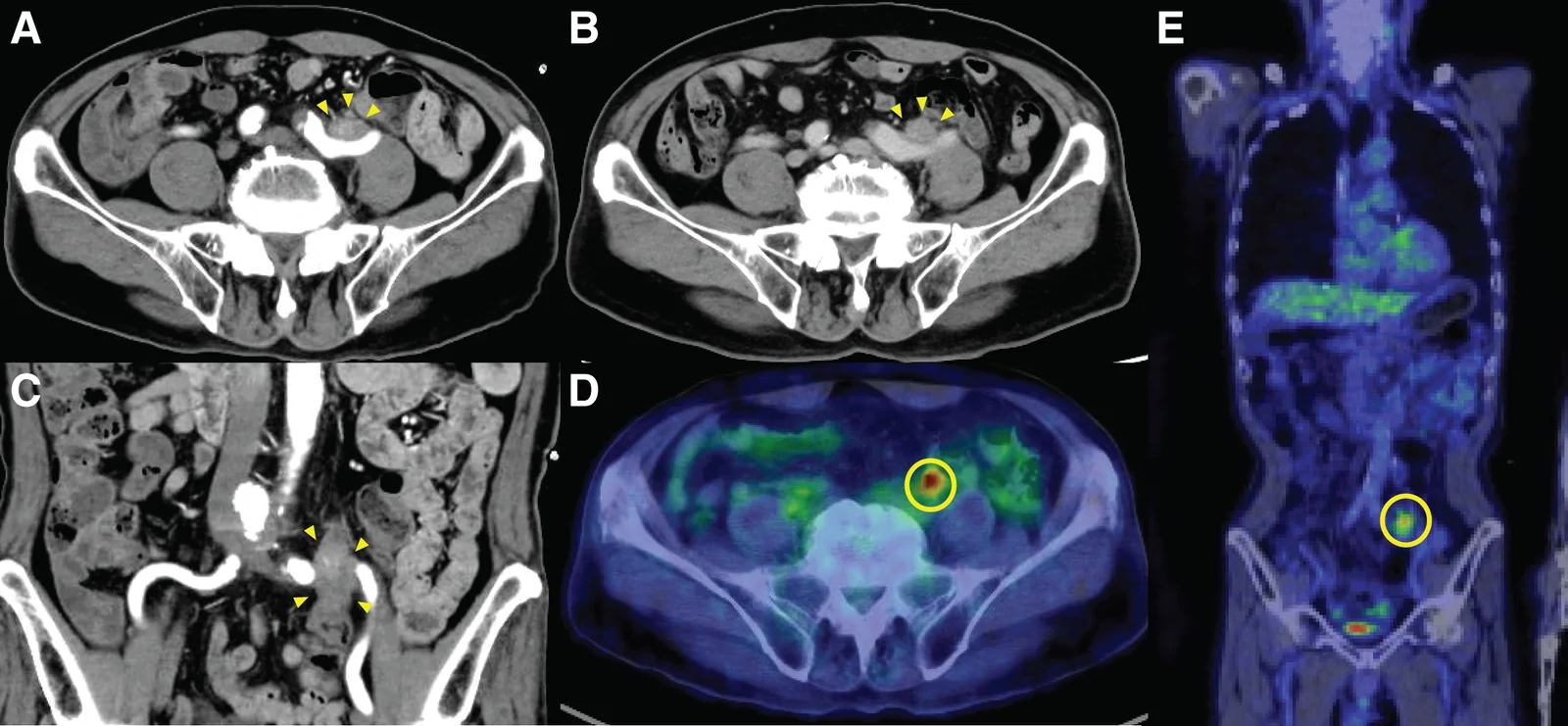
(**A**, **C**) Preoperative contrast-enhanced CT showing interval enlargement to 25 mm without overt iliac vessel invasion. (**B**) Contrast-enhanced CT at the time of initial recurrence detection showing a 20-mm pelvic mass anterior to the left iliac bifurcation. (**D**, **E**) Axial and coronal PET/CT images, respectively, demonstrating focal FDG uptake in the pelvic lesion and no distant metastasis. FDG, fluorodeoxyglucose

Two years after hepatectomy, surveillance imaging revealed a new approximately 20-mm pelvic mass located anterior to the bifurcation of the left common iliac vessels on contrast-enhanced CT (**Fig. 1**). The lesion was suspected to involve the left distal ureter at the level of the common iliac crossing. A left percutaneous nephrostomy tube was placed for left hydronephrosis. The patient was asymptomatic, and serum tumor markers, including CEA and CA19-9, remained within the normal range without interval elevation.

The tumor was RAS-mutant, BRAF wild-type, and microsatellite stable. FOLFOX plus bevacizumab was selected as a standard systemic treatment option for recurrent colorectal cancer with RAS mutation and continued for 3 courses over 2 months, but was stopped because of treatment-related adverse effects before transition to maintenance therapy with bevacizumab and 5-fluorouracil. The lesion enlarged to approximately 25 mm during 4 months of maintenance chemotherapy, consistent with progressive disease (**Fig. 1**). Renal function remained stable throughout this period under nephrostomy management.

Systemic chemotherapy including bevacizumab was discontinued due to treatment-related fatigue, and the case was re-evaluated with curative intent. PET/CT showed increased uptake in the pelvic lesion, but no evidence of other distant metastasis (**Fig. 1**).

Multidisciplinary discussion concluded that the disease remained confined to the pelvis without distant metastasis and that an R0 resection appeared technically feasible based on the absence of major vascular invasion on contrast-enhanced CT. In addition, renal preservation was considered preferable if urinary reconstruction could be performed safely following anticipated ureteral resection. Therefore, laparoscopic curative-intent surgery was planned.

### Surgical procedure

[Fig F2][Fig F3]

**Fig. 2 F2:**
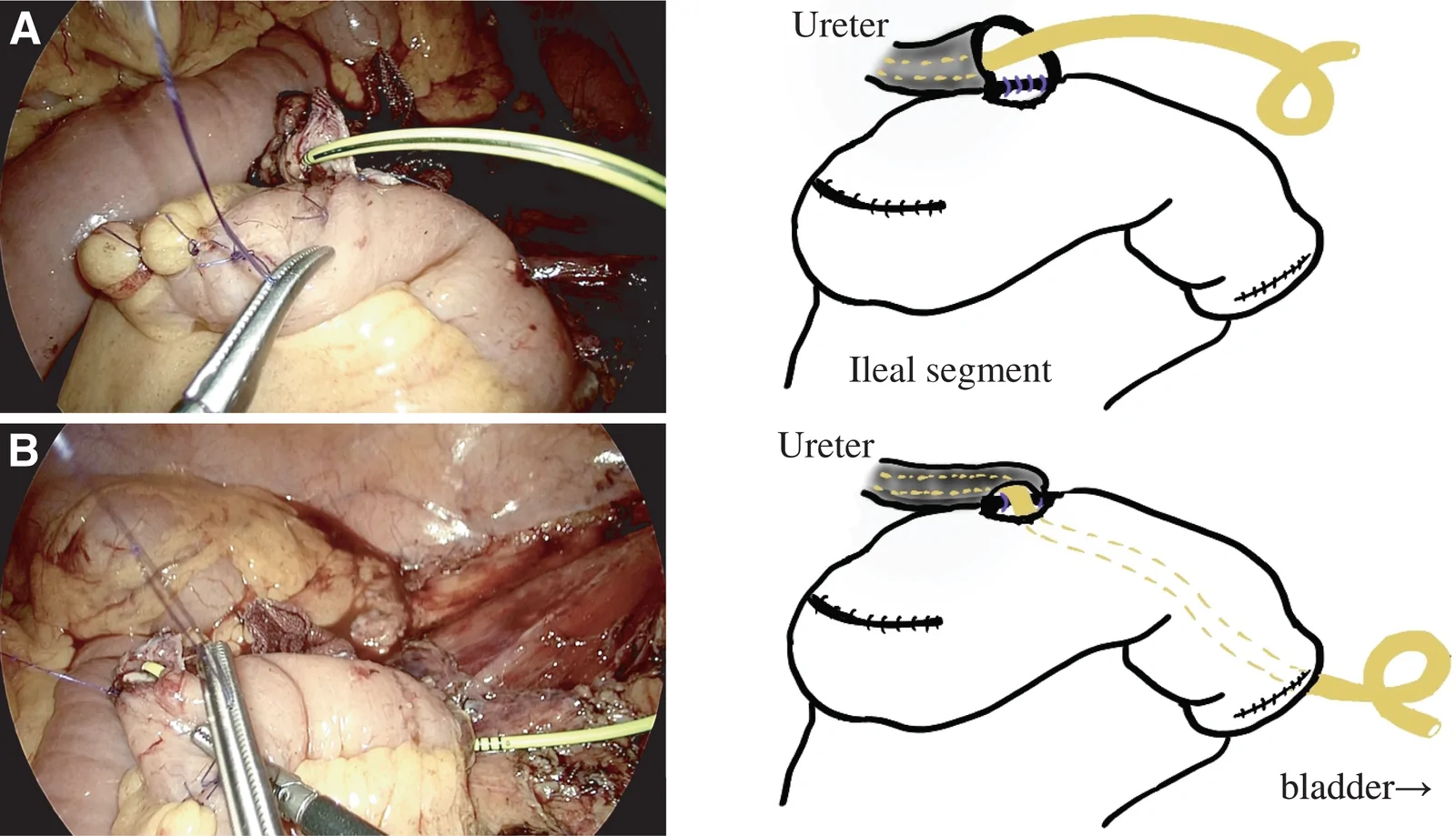
(**A**) The posterior wall was completed with a running 4-0 polyglactin suture. (**B**) A ureteral stent was inserted across the anastomosis and advanced through the conduit. The anterior wall was then completed with a running 4-0 polyglactin suture.

**Fig. 3 F3:**
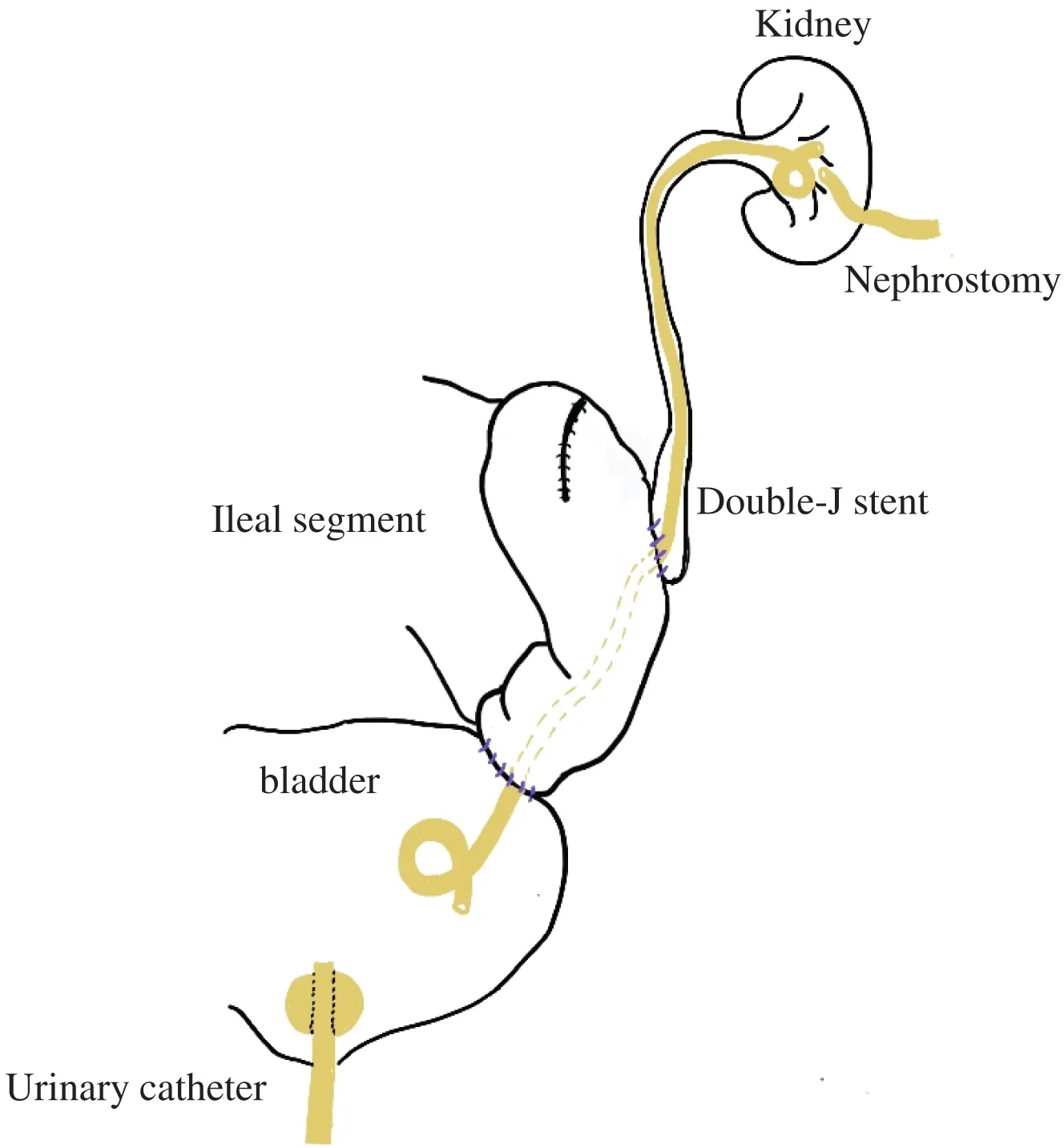
The ileovesical end-to-side anastomosis was performed under direct vision through the Pfannenstiel extraction incision using a single-layer running 3-0 polyglactin suture. A urinary catheter was left in place postoperatively.

1) The patient was placed in the supine position under general anesthesia. A 12-mm umbilical trocar was inserted using an open technique to access the peritoneal cavity. No peritoneal dissemination or intra-abdominal adhesions were observed. Four additional 5-mm trocars were inserted, and the right lower quadrant port was upsized to 12 mm at the end of the operation to allow stapler use for bowel transection.
2) The reconstructed colon was mobilized, and the rectum was dissected cranially beyond the previous anastomosis to the upper part of the Douglas pouch. The recurrent tumor was densely adherent to the left common iliac artery. In case of hemorrhage, vessel control was secured by taping the proximal common iliac artery as well as the external and internal iliac arteries. Although pelvic neural structures required dissection, the vascular adventitia was preserved, and no vascular injury occurred.
3) The left ureter was transected proximal and distal to the tumor and resected en bloc. The gonadal vessels were also resected. The rectum was transected below the peritoneal reflection using a linear stapler with 60-mm and 45-mm cartridges. A transverse suprapubic Pfannenstiel incision was then made, and the colon was additionally divided just proximal to the tumor. A 29-mm circular stapler anvil was placed, and the specimen was extracted. For urinary reconstruction, a 10-cm ileal segment was harvested 20 cm proximal to the terminal ileum. The ileal segment was selected based on sufficient mobility, preservation of the mesenteric vascular pedicle, feasibility of tension-free isoperistaltic interposition, and avoidance of interference with the bowel anastomosis or pelvic operative field. Intestinal continuity was restored using an FEEA. After mobilization of the bladder, an end-to-side ureteroileal anastomosis was created on the side wall of the interposed ileal segment laparoscopically. The ileal segment was oriented isoperistaltically to maintain antegrade urinary flow. The posterior wall was completed with a running 4-0 polyglactin suture. A double-J stent was inserted across the anastomosis and advanced through the interposed ileal segment. The anterior wall was then completed in the same fashion with a running 4-0 polyglactin suture (**Fig. 2**). After colorectal reconstruction, a diverting ileostomy was created. The ileovesical end-to-side anastomosis was performed under direct vision through the Pfannenstiel incision, which had originally been made for specimen extraction. The anastomosis was completed by hand-sewn suturing using a single-layer running 3-0 polyglactin suture over the ureteral stent. A urinary catheter was left in place postoperatively (**Fig. 3**).
4) The total operative time was 441 min, and the estimated blood loss was 102 mL. The urinary reconstruction required approximately 75 min in total. The laparoscopic ureter–ileal anastomosis required approximately 50 min, and the bladder–ileal anastomosis performed through the Pfannenstiel incision required approximately 25 min. The urinary reconstruction was performed by the gastrointestinal surgical team with intraoperative support from urologists.

### Pathological findings

**Fig. 4:**
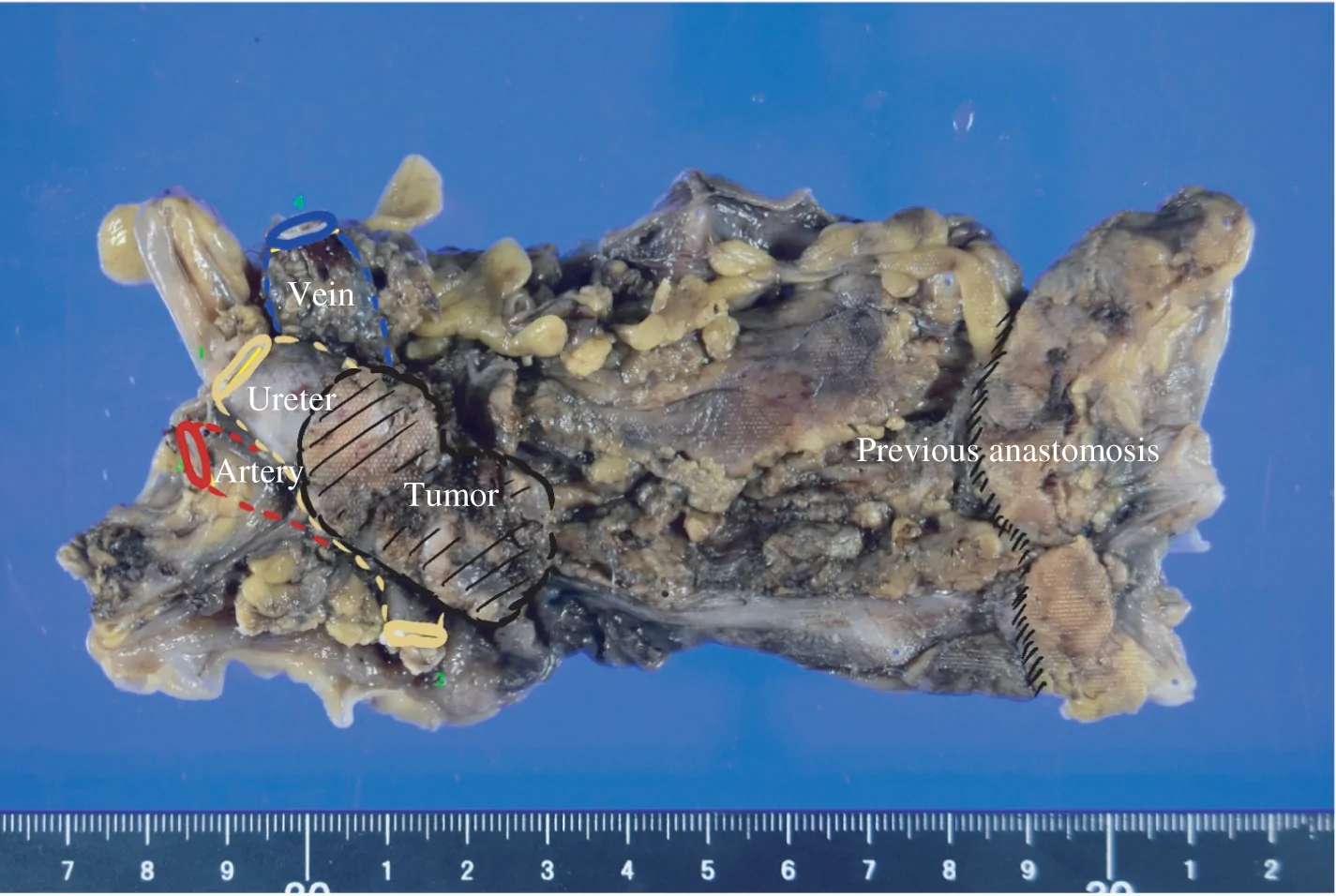
Histopathological examination demonstrated adenocarcinoma consistent with colorectal origin, with invasion of the ureter. The proximal and distal bowel resection margins were negative.

Histopathological examination demonstrated adenocarcinoma consistent with colorectal origin, with invasion of the ureter. The proximal and distal bowel resection margins were negative. Although assessment of the surgical dissection plane was partially limited by thermal artifacts, no microscopic tumor exposure was identified on the dissected surface. Therefore, the final resection status was regarded as pathologically R0 (**Fig. 4**).

### Postoperative course

**Fig. 5:**
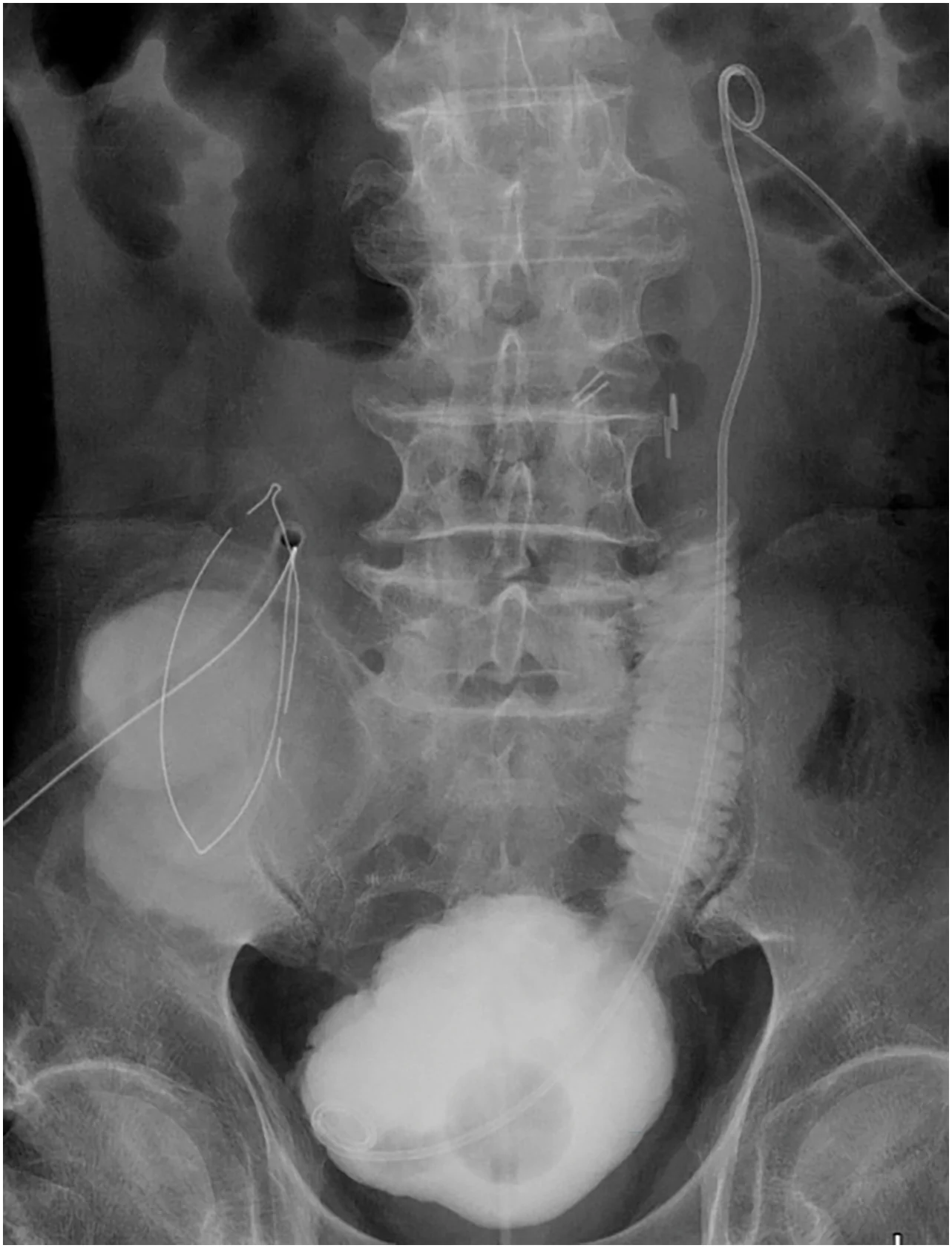
A cystogram performed through the urinary catheter showed no leakage on POD 8.

Postoperative paralytic ileus occurred and resolved conservatively (Clavien–Dindo grade II). A cystogram performed through the urinary catheter showed no leakage, and the catheter was removed on POD 8 (**Fig. 5**). The patient was discharged on POD 18. The double-J stent was removed on POD 32, and the percutaneous nephrostomy tube was removed on POD 49.

The patient had pre-existing chronic kidney disease; however, renal function was preserved after surgery without obvious deterioration. The serum creatinine level and estimated glomerular filtration rate were 1.42 mg/dL and 37 mL/min/1.73 m^2^ before surgery, 1.49 mg/dL and 36 mL/min/1.73 m^2^ after surgery, and 1.48 mg/dL and 36 mL/min/1.73 m^2^ at the latest follow-up, respectively.

No adjuvant therapy was administered. Radiotherapy for possible margin involvement was discussed but was not performed because of technical constraints related to the limited pelvic target volume and prior bevacizumab exposure. Follow-up consisted of tumor marker measurement and CT. The patient remained free of clinical and radiological recurrence 12 months after salvage surgery.

## DISCUSSION

Surgery remains central to curative-intent management of LRRC when complete resection is anatomically feasible, because local control and long-term survival are strongly influenced by resection status.^3)^ In practice, however, LRRC surgery is frequently complicated by dense fibrosis and altered planes after previous surgery, and recurrence may extend to the pelvic sidewall, ureter, or iliac vessels, increasing the risk of major bleeding and postoperative morbidity.^1)^ Accordingly, meticulous preoperative assessment of resectability, including evaluation of vascular involvement and the anticipated extent of multivisceral resection, is essential to align surgical aggressiveness with oncologic benefit.

In the present case, assessment of the surgical dissection plane was partially limited by thermal artifacts; however, no microscopic tumor exposure was identified, and the final resection status was regarded as pathologically R0. This finding underscores the inherent difficulty of margin assessment in salvage pelvic surgery.

When LRRC involves the ureter, the reconstructive strategy after oncologic ureterectomy should be individualized according to the anticipated defect length, tissue mobility, and the feasibility of a tension-free distal reimplantation. For short distal defects, ureteroneocystostomy (e.g., psoas hitch or Boari flap) can be appropriate. However, in the salvage setting after prior rectal surgery, dense pelvic adhesions and scarring may markedly limit ureteral and bladder mobility.^4)^ In the present case, although the bladder was not involved, the lesion was located at the level of the common iliac crossing, and the expected distance and limited mobility raised concern that direct ureteroneocystostomy would be under tension and therefore prone to leakage or stricture. Accordingly, ileal ureter interposition was selected to enable en bloc ureterectomy while maintaining urinary continuity and preserving renal function.

Compared with urinary diversion, a renal-preserving reconstruction may reduce the long-term lifestyle burden associated with a permanent urinary stoma. Ileal ureter replacement has been reported to provide acceptable long-term patency with preserved renal function in properly selected patients, albeit with recognized risks such as mucus production, urinary tract infection, ureteroenteric stricture, and metabolic acidosis.^5)^

Large clinical series have described stable renal function in most patients and relatively low rates of late strictures and fistulae, supporting the overall validity of the procedure when meticulous technique and follow-up are ensured.^6,7)^ From an operative standpoint, keeping the ileal segment as short as feasible, preserving mesenteric blood supply, maintaining an antegrade orientation, and constructing watertight anastomoses are key technical principles to mitigate these risks.^6)^ Perioperatively, temporary urinary drainage with a ureteral stent (and nephrostomy when present preoperatively) facilitates protected healing and allows staged confirmation of patency before device removal; continued surveillance of renal function and hydronephrosis is essential to detect early obstruction or infection.^5)^

A laparoscopic case has been reported for primary sigmoid colon cancer with ureteral involvement and ileal interposition^8)^; however, no report of laparoscopic salvage surgery for LRRC requiring urinary tract resection and reconstruction has been identified to the best of our knowledge. A potential advantage of laparoscopy is reduced abdominal wall trauma, resulting in smaller incisions and potentially less wound-related morbidity.^9)^ Conversely, a recognized drawback is prolonged operative time compared with open surgery; however, because the pelvic dissection required for LRRC often overlaps with the technical domain of total pelvic exenteration, a laparoscopic approach may be feasible in selected patients when performed by surgeons with sufficient expertise in advanced minimally invasive pelvic surgery.^10)^

In a large series of ileal ureter replacement, fistula occurred in 6.6% of patients.^6)^ Risk factors for ureteroileal anastomotic leakage include compromised ureteral blood supply due to excessive ureteral mobilization, tension at the anastomosis, and prior pelvic radiotherapy.^11,12)^ Anti-vascular endothelial growth factor (VEGF) therapy with bevacizumab has been associated with impaired wound healing and an increased risk of postoperative wound-related complications.^13)^ Accordingly, an adequate washout period before elective surgery is generally recommended; reviews in colorectal cancer populations have suggested that perioperative serious adverse events are low when a time-to-surgery interval of approximately 5–6 weeks is respected, and many expert recommendations favor ≥6 weeks in the setting of major abdominal surgery.^14)^

In the present case, the last administration of bevacizumab was on January 6, 2025, and surgery was performed on March 13, 2025 (approximately 9 weeks after the final dose), which is consistent with these commonly recommended intervals. Given that our procedure involved both colorectal anastomosis and urinary reconstruction, careful perioperative management was emphasized, including tension-free anastomoses, meticulous tissue handling, and close postoperative surveillance for leakage or delayed healing.

## CONCLUSIONS

Laparoscopic salvage resection with en bloc ureterectomy and ileal ureter interposition may be a feasible renal-preserving option for selected patients with ureter-invading LRRC. Multidisciplinary collaboration and meticulous technique are essential, and further cases with longer follow-up are warranted.

## DECLARATIONS

### Availability of data and materials

The datasets supporting the conclusions of this article are included within the article.
